# Deregulation of Interferon Signaling in Malignant Cells 

**DOI:** 10.3390/ph3020406

**Published:** 2010-02-04

**Authors:** Efstratios Katsoulidis, Surinder Kaur, Leonidas C. Platanias

**Affiliations:** Robert H. Lurie Comprehensive Cancer Center and Division of Hematology-Oncology, Northwestern University Medical School and Jesse Brown Veterans Affairs Medical Center, Chicago, IL 60611, USA

**Keywords:** interferon, signaling pathways, cancer

## Abstract

Interferons (IFNs) are a family of cytokines with potent antiproliferative, antiviral, and immunomodulatory properties. Much has been learned about IFNs and IFN-activated signaling cascades over the last 50 years. Due to their potent antitumor effects *in vitro* and *in vivo*, recombinant IFNs have been used extensively over the years, alone or in combination with other drugs, for the treatment of various malignancies. This review summarizes the current knowledge on IFN signaling components and pathways that are deregulated in human malignancies. The relevance of deregulation of IFN signaling pathways in defective innate immune surveillance and tumorigenesis are discussed.

## 1. Introduction

**Figure 1:**
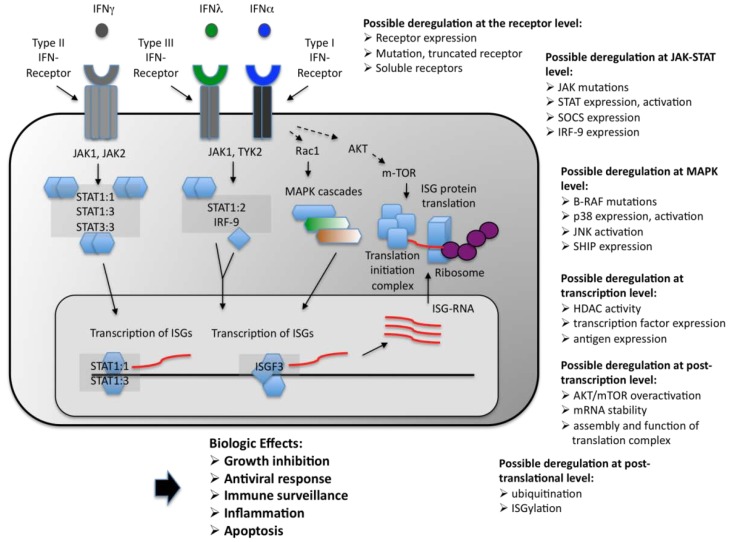
Simplified depiction of Type I, II and III interferon signaling events and outcomes in normal, IFN sensitive cells. Possible deregulation of various IFN signaling components in malignant and IFN resistant cells is also shown.

Interferons (IFNs) were discovered and named by Isaacs and Lindenmann in 1957 due to their ability to interfere with the effects of viral infection of cells [1]. Extensive studies over the years have shown that IFNs are pleiotropic cytokines secreted by several cell types as part of the innate immune response [2,3,4,5]. Once IFNs bind to their respective receptors, they trigger many signaling pathways that modulate a wide range of biological responses including antiviral, growth regulatory, immunomodulatory, and pro-apoptotic cellular effects [[6,7,8]. Due to their important functions, IFNs and their signaling pathways have been under intensive investigation over the past two decades [9,10]. It is now well established that several signaling pathways and their downstream effectors are required for IFN-dependent biological responses [6,7,11,12,13,14,15]. As IFNs are potent suppressors of growth, tumor cells with deleted, suppressed or deregulated IFN signaling components have a proliferation advantage, by evading the effects of endogenous IFN and the innate immune response. Such a view is supported by studies showing that cells with defects Type I and Type II IFN signaling components are more susceptible to spontaneous and induced tumor formation [16,17,18,19,20,21] and by studies showing that many malignancies are associated with deregulated/defective IFN-signaling cascades [19,20,21,22,23,24,25,26,27] (Figure 1).

## 2. Classification of the IFNs

IFNs are grouped based on their sequence homology and receptor binding into three major groups: Type I IFNs (α, β, δ, ε, κ, τ, ω) which use the Type I IFN receptor [3,4], Type II IFNs (γ) which use the Type II IFN receptor [3,4] and a third, recently identified type of IFNs, IFNλ, which uses an IFNλ-specific receptor [28]. There are several subtypes of IFNs: IFNα proteins are encoded by as many as 14 genes in humans [29], IFNλ has three subtypes [28] while IFNβ and IFNγ proteins are encoded by single genes [30,31]. In addition to the naturally occurring IFNs, chemically modified IFNs, such as pegylated IFNs have been in use in clinical medicine due to better pharmacologic properties and/or superior bioavailability [32,33]. 

## 3. The IFN Receptors in Malignancies

The Type I IFN receptor consists of the interferon alpha receptor (IFNAR) 1 and IFNAR2 subunits [3,4]. During engagement by Type I IFNs, these two major chains for the receptor mediate activation of receptor-associated Janus kinase (JAK) 1 and TYK2 tyrosine kinases [6,7,34,35]. The phosphorylated JAK1 and TYK2 kinases activate multiple signaling cascades, as discussed below. Similarly, the Type II IFN receptor consists of the interferon gamma receptor (IFNGR) 1 and IFNGR2 subunits, which associate with JAK1 and JAK2 [34,35], respectively, which in turn activate the Type II IFN-dependent JAK-STAT pathways to generate IFNγ responses [7,36,37]. The recently described Type III IFN, IFNλ, binds to a different receptor, which consists of the IFNλR1 and IL10R2 receptor chains and transduces signals *via* the JAK1 and TYK2 receptor tyrosine kinases [28,38,39,40,41]. Interestingly, there appears to be considerable regulation of IFN signaling at the receptor level: Type I IFN receptor expression has been shown to be induced in response to viral infection of host cells [42] and single nucleotide polymorphisms (SNPs) in the promoter region of IFNR- encoding genes IFNAR2 and IL10RB have been found to be associated with hepatitis B viral persistence [43]. Furthermore, SNPs in the promoter regions of IFNAR1 and IFNAR2 affect susceptibility to multiple sclerosis [44] and responsiveness to IFN treatment [42,43,44]. There is also evidence for the existence of a truncated, soluble IFN receptor, sIFNAR2a, found in body fluids such as serum and peritoneal fluid in humans and mice [45]. The functional relevance of such soluble receptors is not fully established and there is evidence for both positive and negative contributions of such receptors in the generation of the effects of IFNs [35,45]. 

The role of deregulated IFN receptor expression in tumor development and expression is unclear. IFNAR1 was recently shown to be degraded in malignant melanoma cells with activated BRAF mutation *via* a mechanism, which involves β-Trcp ubiquitin ligase [46]. Moreover mice with defective Type I and Type II IFN receptors develop mutagen-induced tumors more frequently than their normal counterparts [17,18,47]. On the other hand, no correlation could be found between expression of IFN receptors and the metastatic ability of renal cell carcinoma (RCC) cells [48]. One possibility is that deregulation of IFN receptor expression is a temporary effect and only necessary during the initial evasion of the tumor surveillance response. It is also possible that other signaling components, such as JAK-STAT, AKT and mitogen-activated protein kinase (MAPK) pathways may be ‘preferentially’ deregulated since these pathways regulate signals of a broader array of cytokines and growth factors [49]. 

## 4. The JAK-STAT Pathway

There are several known members of the JAK and STAT (signal transducer and activator of transcription) family of proteins. As mentioned above, JAKs are receptor-associated protein tyrosine kinases, which are activated upon ligand binding to the IFN receptors, and subsequently bind and phosphorylate STAT proteins which in turn form complexes that participate in the regulation of IFN mediated gene transcription [6,7,15]. In addition to the classical JAK-STAT signaling mechanisms, other mechanisms involving unphosphorylated STATs and/or NFkB signaling components have also been identified [50,51]. 

### 4.1. JAK kinases in Malignancies

Four members of the JAK family of kinases are known: JAK1, JAK2, JAK3 and TYK2 [6,7,34,52]. JAK kinases are relatively large-size proteins (120–140 kD) and have a common domain structure, consisting of a Band4.1, Ezrin, Radixin and Moiesin (FERM) domain, the phosphotyrosine-binding SH2 domain, as well as regulatory pseudokinase and kinase domains [53,54]. The FERM domain is involved in receptor association and also participates in the regulation of kinase activity [53,54]. JAK1 is essential for Type I and Type II IFN signaling [6,7,14,15,35], while JAK2 mediates Type II IFN signaling [36,37]. TYK2, which was the first JAK kinase identified, also associates with the Type I IFNR, but surprisingly, cells from TYK2 knockout mice show only partial defects in antiviral responses to IFNα [55,56]. In contrast to the other JAKs, JAK3 is selectively expressed in leukocytes where it associates with the IL-2 receptor (IL-2R) γ-chain and participates in signaling required for lymphoid activity [57,58,59]. JAK3 does not play a role in IFN signaling, since none of the known IFNs induces JAK3 activation [6,7,57,58,59]. Interestingly, abnormal JAK3 expression and constitutive activation has been identified in human colon and lung carcinoma cell lines, as well as acute megakaryoblastic leukemia cells [60,61,62]. JAK1 mutations seem to be less frequent, though gain-of-function mutations have been identified in isolated cases of acute myeloid leukemia [63] and in T-cell acute lymphoblastic leukemia (T-ALL) patients [64]. The most commonly mutated JAK family member in myeloproliferative disorders and acute leukemia is JAK2 [25,65]. JAK2 is constitutively activated either *via* fusion to other proteins, such as in translocation Ets leukemia (TEL)-JAK2, pericentriolar material 1 (PCM1)-JAK2 and breakpoint cluster region (BCR)-JAK2 or *via* activating amino acid substitutions or deletions [25,66]. An interesting twist is presented by a new study, which found that JAK2 translocates to the nucleus where it phosphorylates histone H3, thereby promoting gene transcription and expression of the hematopoietic Imo2 oncogene [67,68]. In contrast to JAK2, much less is known about TYK2 mutations in human malignancies. However, inactivation of TYK2 by a mutation leading to early termination of TYK2 mRNA expression and several JAK3 inactivating amino acid substitutions have been linked to immunodeficiency and SCID [25,34,69].

### 4.2. STAT Proteins in Malignancies

The family of signal transducers and activators of transcription consists of seven members: STAT1, STAT2, STAT3, STAT4, STAT5a, STAT5b and STAT6 [6,34,70]. Human STAT proteins have a conserved domain structure, which consists of an N-terminal domain, a coiled coil domain, a DNA binding domain, as well as Src homology 2 (SH2) and transactivation domains [34,70,71]. STAT proteins are phosphorylated and activated in response to several cytokines, growth factors and hormones [6,34,70]. IFNR binding results in tyrosine phosphorylation of STAT1 (Type I, Type II and Type III IFNs), STAT2 (Type I, Type III IFNs), STAT3 (Type I, Type II IFNs), STAT4 (Type I IFNs) and STAT5 (Type I IFNs) [6,7,15,38,71]. Serine phosphorylation of STAT1 in response to Type I IFNs has also been described, and protein kinase C (PKC)δ has been shown to be the responsible serine kinase [72]. Phosphorylated STAT proteins accumulate in the nucleus *via* an importin mediated pathway, where they bind to specific sequences in the promoters of target genes, to regulate their transcription [6,7,15,71,73,74]. Interestingly, unphosphorylated or only serine-phosphorylated STAT1 and STAT3 proteins have also been shown to regulate gene transcription of certain groups of genes [50]. The regulation of Type I and Type III IFN dependent gene transcription involves IRF-9 (also known as p48) which forms the ISGF3 complex with tyrosine-phosphorylated STAT1 and STAT2, and specifically binds and regulates interferon-stimulated response element (ISRE) dependent gene expression [6,7,14,15]. Type II IFN regulated gene transcription on the other hand, involves the activation and dimerization of STAT1 and STAT3 proteins, which form DNA-binding SIE complexes [6,7,14,15]. IFNs also induce STAT5, CrkL and other transcriptional regulators [6,7]. After their release from DNA, STATs are de-phosphorylated and exit the nucleus *via* an incompletely understood mechanism [50,71,75]. It is thought that STAT dephosphorylation exposes a nuclear export sequence, which is hidden in phosphorylated STAT dimers [75,76]. 

Important roles for constitutively active STAT5 and STAT3 in the pathogenesis of various malignancies have been previously demonstrated [49,77,78,79,80]. In contrast, STAT1 activation is linked to generation of antiproliferative effects, while STAT1 deficiency results in immune-suppression, in part *via* decreased antigen expression [17,34,81]. STAT1 knockout mice are highly susceptible to bacterial infection [82,83] and develop increased numbers of chemically induced and spontaneous tumors compared to parental mice [17,18]. Consistent with these findings, fibroblasts from STAT1 knockout MEFs grow faster than their wild type counterparts, but IFN mediated growth inhibition is not completely blocked in the absence of STAT1 [34,84]. STAT1 phosphorylation and interferon stimulated gene (ISG) expression was recently analyzed in lymphocyte populations from breast cancer, melanoma and gastrointestinal cancer patients and compared to their normal counterparts for signaling in response to Type I and Type II IFNs [27]. Interestingly, IFN-signaling was suppressed in peripheral lymphocyte populations from patients harboring any of the three malignancies, evidenced by significantly reduced levels of activated STAT1 and the decreased expression of several ISGs, raising the intriguing possibility of a global suppression of the IFN system in various cancers [27].

The simultaneous activation of various STAT proteins by IFNs seems puzzling. It is plausible that, in order to strike a balance between anti- and pro-proliferative signaling effects, different cells activate pro-apoptotic as well as anti-apoptotic genes. Interestingly, IFN-regulated STAT-dependent gene transcription is further regulated *via* crosstalk with other auxiliary signaling pathways, such as the p38 MAPK pathway [7,11,13,85,86,87,88], allowing cells to optimally respond to IFN treatment. Treatment of IFN responsive CML cell lines and primary cells has been found to result in activation of JAK-STAT and auxiliary signaling pathways, ISG expression and growth suppression of early leukemic progenitor cells [13,88]. It is possible that IFN signaling components compete with pathways also utilized by the BCR-ABL oncogene, such as STAT3, STAT5 or the CrkL adaptor protein. On the other hand, wild type BCR-ABL, as well as STI-571 resistant BCR-ABL transformed BA/F3 cells appear to have defective IFN dependent gene transcription, due to impaired STAT1, STAT3 and p38-MAPK phosphorylation [89]. In accordance with these findings, generation of IFN-dependent antiviral and antiproliferative responses is defective in BCR-ABL transformed cells, suggesting a selection against the IFN system [89]. This concept is further supported by the suppression of other growth inhibitory targets used by IFNs in BCR-ABL expressing cells, such as p38 MAPK and IRF-1 [22,90,91,92]. 

Another important mechanism by which IFNs mediate their biologic effects is ISG15 expression, a STAT-regulated gene, and subsequent ISGylation of various proteins within target cells [93,94]. Surprisingly, the ISG15 gene/protein was recently found to be up-regulated in breast cancer cells and correlate with unfavorable prognosis [95]. In contrast, ISGylation enzymes such as UBE1L, have been found to be suppressed or deleted in small lung carcinoma cells and leukemia cell lines such as K562 CML cells [93,96,97]. Consistent with a tumor suppressor role for the ISG15 system, various melanoma cell lines have increased levels of Ubp48, a ISG15 deconjugating enzyme [98].

## 5. Additional Regulatory Mechanisms and Concluding Remarks

There are several auxiliary pathways and different levels of regulation that participate in the generation of the antiproliferative and antiviral effects of IFNs, including MAPK signaling cascades [7,11,85,86,87,88], the PI3’K-AKT pathway [49,99,100,101], PKC pathways [102], SOCS proteins [103], and HDACs [12]. However, the potential existence and relevance of defects in such pathways in IFN-resistance in malignant cells remains to be examined. Although a lot still remains to be learned regarding the mechanisms of action of IFNs, the multiplicity of pathways activated by IFNs and the requirement for coordination of various signals for the generation of antineoplastic effects provides several potential therapeutic target points for the development of novel anti-cancer drugs. Based on this, one can assume with a certain degree of certainty that research in the broad field of IFNs will continue to lead to important therapeutic developments and breakthroughs in the near and distant future. 
